# Relationship between presarcopenia and event occurrence in patients with primary hepatocellular carcinoma

**DOI:** 10.1038/s41598-020-67147-7

**Published:** 2020-06-23

**Authors:** Hitomi Takada, Fumitake Amemiya, Tomoki Yasumura, Hiroki Yoda, Tetsuya Okuwaki, Naoto Imagawa, Naruki Shimamura, Keisuke Tanaka, Makoto Kadokura, Shinya Maekawa, Nobuyuki Enomoto

**Affiliations:** 1 Department of Gastroenterology and Hepatology, Municipal Hospital of Kofu, Yamanashi, Japan; 2 First Department of Internal Medicine, Faculty of Medicine, University of Yamanashi, Yamanashi, Japan

**Keywords:** Gastroenterology, Oncology

## Abstract

Presarcopenia is a prognostic factor in patients with hepatocellular carcinoma (HCC). The Japan integrated staging (JIS) score is a prognostic method that combines the Child–Turcotte–Pugh classification and the tumor-node-metastasis (TNM) staging for HCC. We investigated the relationship between presarcopenia, the JIS score, and prognosis in patients with primary HCC. This retrospective study included 153 patients with primary HCC who were hospitalized from October 2011 to March 2018 at Municipal Hospital of Kofu. The skeletal muscle mass was measured using simplified psoas muscle mass index (PMI) based on CT imaging, and PMI using the volume analyzer SYNAPSE VINCENT ver3.0. We diagnosed presarcopenia based on the cut off value according to the assessment criteria for sarcopenia in liver disease defined by the Japan Society of Hepatology. Forty-three patients (28%) were diagnosed with presarcopenia. The median event-free survival was significantly worse in patients with presarcopenia than those without presarcopenia (*P* = 0.016). In multivariate analysis, presence of presarcopenia, JIS score ≥3, alpha-fetoprotein ≥200 ng/ml, and prothrombin induced by vitamin K absence-II ≥ 200 mAU/ml were significant prognostic factors. Among the patients with JIS scores ≥3, there was no difference in the event occurrence rate with presence of presarcopenia (*P* = 0.96). Among the patients with JIS scores ≤2, the median event-free-survival was significantly shorter in those with presarcopenia than those without presarcopenia (*P* = 0.045). Presarcopenia was an independent prognostic factor in patients with primary HCC. In patients with JIS scores ≤2, the median event-free survival was significantly shorter in those with presarcopenia compared to those without presarcopenia. In the patients with JIS scores ≥3, there was no difference in the event occurrence rates in those with and without presarcopenia.

## Introduction

Sarcopenia is characterized by low skeletal muscle mass^1^, skeletal muscle weakness, and decreased physical performance, first proposed by Rosenberg *et al*. in 1989^2^. Sarcopenia is a core symptom of frailty, a poor prognostic factor in the elderly that has garnered increasing attention in recent years. The diagnostic criteria of sarcopenia are defined by several groups including the European Working Group on Sarcopenia in Older People, the International Working Group on Sarcopenia, and Asia Working Group on Sarcopenia^3^. Low skeletal muscle mass and muscle weakness, defined by a decrease in grip strength or walking speed, are necessary for diagnosis by all standards. Conversely, according to the assessment criteria for sarcopenia in liver disease defined by the Japan Society of Hepatology^1^, patients with chronic liver disease who had decreased grip strength and low muscle mass by computed tomography (CT) or bioelectrical impedance analysis (BIA) are defined to exhibit sarcopenia. The prognosis of patients with sarcopenia is worse than those without sarcopenia among patients with liver cancer^4^. Additionally, patients with low muscle mass in the absence of muscle weakness are defined to have presarcopenia, which was also reported to be a prognostic factor independent from liver function and staging in patients with liver cancer^5–8^. Importantly, presarcopenia can be improved by therapeutic interventions including diet, exercise, and medications, with many studies published in recent years^9,10^; therefore, prognosis can be potentially improved by treatment of presarcopenia. However, the characteristics of patients with liver cancer cases in whom prognosis might be aggravated by the presence of sarcopenia remain unclear.

The Japan integrated staging (JIS) score, a prognostic score for patients with liver cancer that combines the Child–Turcotte–Pugh classification and the tumor-node-metastasis (TNM) staging, was first reported by Kudo *et al*. in 2003^11^. The JIS score is a predictive score that is convenient for use in clinical settings. In the current study, we retrospectively examined the relationship among presarcopenia, JIS score, and prognosis in patients with primary hepatocellular carcinoma (HCC) to clarify the characteristics of patients in whom that presence of presarcopenia is associated with prognosis.

## Methods

### Subjects

This was a retrospective cohort study of 153 patients with primary HCC patients who were hospitalized from October 2011 to March 2018 at Municipal Hospital of Kofu. This study complied with the Declaration of Helsinki and was approved by the institutional ethics committee (ethics committee for clinical studies of Municipal Hospital of Kofu: Rinshoukenkyu-Rrinrishinsa-Iinkai (in Japanese), approval number 31-2). Written, informed consent was obtained from each patient. The diagnosis of HCC was based on pathological assessment or imaging. Diagnostic imaging was performed using dynamic CT, Gadoxetic acid-enhanced magnetic resonance imaging, or contrast-enhanced ultrasound with Sonazoid. HCC was defined based on classical findings including hypervascularity in the arterial phase and washout in the portal phase. Patients with an Eastern Cooperative Oncology Group performance status score of 3 or more, those with primary cancers in other organs, and those with a follow-up period of one month or less from discharge were excluded from this study.

### Muscle mass measurement

The gold standards for the measurement of skeletal muscle mass index are CT or BIA, both of which require special software and equipment. There is no special software or equipment to evaluate the muscle mass in out hospital. We have used 1) simplified psoas muscle mass index (PMI) based on CT imaging, and 2) PMI using the volume analyzer SYNAPSE VINCENT ver3.0. In this study, we evaluated muscle mass by the above two measurement methods, and the patients with low muscle mass by either measurement method were diagnosed presarcopenia. Simplified PMI, a simple method for measuring muscle mass that can be used in a clinical setting^12^, is calculated by dividing the sum of the long axis and short axis lengths of the intestinal psoas muscles on both sides of the L3 vertebra by the square of body height using CT images. In the current study, PMI and simplified PMI were measured using CT images obtained within 1 month before and after hospitalization.

### Diagnosis of low muscle mass

The cutoff value for simplified PMI was 6.0 cm^2^/m^2^ for males and 3.4 cm^2^/m^2^ for females according to the assessment criteria for sarcopenia in liver disease published by the Japan Society of Hepatology^1,8^. The cutoff value for PMI was 6.36 cm^2^/m^2^ for males and 3.92 cm^2^/m^2^ for females^12^. Measurements of muscle mass and diagnosis of low muscle mass were performed by two hepatology specialists with experience in muscle mass measurement.

### Event occurrence

Event-free survival was calculated starting from the date of hospitalization, with the date of event occurrence as the end point. The following parameters were evaluated: age; sex; laboratory data including albumin, bilirubin, creatinine, gamma-glutamyl transferase (γ-GTP), platelet, prothrombin time, Child–Pugh grade, alpha-fetoprotein (AFP), lens culinaris agglutinin-reactive fraction of AFP, and prothrombin induced by vitamin K absence-II (PIVKA-II); HCC status including maximum tumor size, number of tumors, vascular invasion, distant metastasis, and TNM classification; and the presence of presarcopenia.

Event occurrence date was defined as the date of death or the date of next hospitalization. Events requiring the next hospitalization were ascites, encephalopathy, gastrointestinal bleeding, infection, bone fracture, deterioration of the performance status, and cancer development in other organs. Cases of HCC recurrence were excluded in this study.

### Statistical analysis

Fisher’s test was used for categorical variables. Normal distribution of the data was evaluated using the Kolmogorov-Smirnov test, and unpaired Student’s *t* test was used for continuous variables. Data were presented as means ± standard deviation. The Kaplan-Meier curves and the log-rank test were used for event-free survival, and the Cox proportional hazards model was used for the assessment of the event-free survival-associated factors. P values of < 0.05 were considered to indicate significance. The best cut-off values in receiver operating characteristic (ROC) analysis were determined by Youden index. All statistical analyzes were performed using EZR (Saitama Medical Center, Jichi Medical University, Saitama, Japan), a graphical user interface for R (The R Foundation for Statistical Computing, Vienna, Austria). More precisely, it is a modified version of the R commander designed to include statistical functions frequently used in biostatistics^13^.

## Results

### Study cohort

The background characteristics of the cohort patients are shown in Table 1. The mean age was 73 ± 9.2 years, and there were 114 (75%) male patients. The cohort comprised 93 (62%), 46 (29%), and 14 (9%) patients in Child–Pugh grades A, B, and C. According to the TNM classification, 26 (17%), 62 (41%), 32 (21%), and 33 (21%) patients were stage 1, 2, 3, and 4. Furthermore, 15 (10%), 56 (37%), 34 (22%), 25 (16%), 17 (11%), and 6 (5%) had JIS scores of 0, 1, 2, 3, 4, and 5, respectively. Presarcopenia was confirmed in 43 cases (28%).

**Table 1:** Backgrounds of patients.

	n = 153 (%)
Age: years, mean ± SD	73 ± 9.2
Male: n (%)	114 (75)
Etiology: virus/alcohol/NASH/others	100/19/33/1 (65/12/22/1)
Body mass index, mean ± SD	22 ± 3.4
Complicated with diabetes: n (%)	44 (29)
Maximum size of HCC nodule in the liver: mm, median (range in IQR)	35 (18–68)
Number of HCC nodules: 1/2/3/4 or more, n (%)	84/25/14/31 (54/16/9/21)
Presence of ascites: n (%)	30 (20)
Presence of hepatic encephalopathy: n (%)	8 (5.2)
Child-Pugh grade: A/B/C: n (%)	93/46/14 (62/29/9)
HCC stage: 1/2/3/4, n (%)	26/62/32/33 (17/41/21/21)
JIS score: 0/1/2/3/4/5, n (%)	15/56/34/25/17/6 (10/37/22/16/11/5)
Albumin: g/dl, mean ± SD	3.6 ± 0.62
Total bilirubin: g/dl, mean ± SD	1.7 ± 2.8
γ-GTP: U/l, mean ± SD	142 ± 194
Prothrombin time: %, mean ± SD	82 ± 20
AFP: ng/ml, median (range in IQR)	33 (6.2–177)
AFP L3 index: %, median (range in IQR)	7.8 (2.1–44)
PIVKA-α: mAU/ml, median (range in IQR)	117 (23–3675)
Presence of presarcopenia: n (%)	43 (28)

Continuous values are expressed as mean ± standard deviation. NASH; nonalcoholic steatohepatitis, BMI; body mass index, HCC; hepatocellular carcinoma, ALT; alanine aminotransferase, AFP; alpha-fetoprotein, AFP L3 fraction; Lens culinaris agglutinin-reactive fraction of AFP, PIVKA-II; prothrombin induced by vitamin K absence II.

### Event occurrence

**Figure 1:**
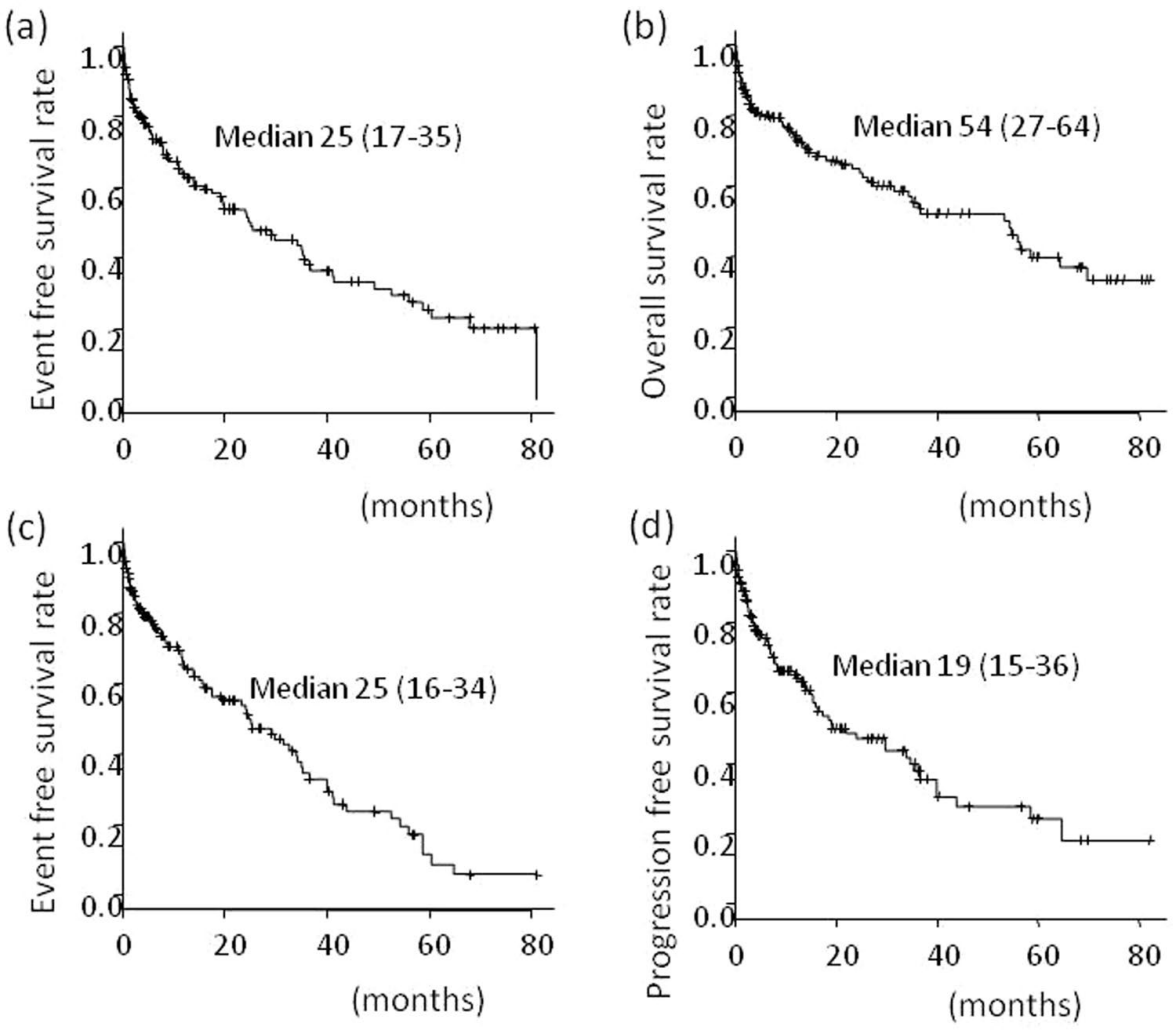
(**a**) Event-free survival (all events necessary for hospitalization), (**b**) overall survival, (**c**) event-free survival (events related to cirrhosis), (**d**) progression-free survival in all 153 patients.

The median observation period was 13.9 (2–86) months, and events requiring hospitalization except HCC recurrence occurred in 79 cases (52%). The median event-free survival was 25.0 (17.4–35.4) months (Fig. 1a). The rates of events at 12, 36, and 60 months were 35%, 62%, and 75%, respectively. These events included death, infection, worsening of the performance status, gastrointestinal tract bleeding, encephalopathy, fractures, ascites, cancer development in other organs, and other events in 29, 12, 11, 9, 4, 4, 3, 3, and 4 patients, respectively. The median survival time, median time to cirrhosis-related events, and median progression-free survival were 54 (27–64), 25 (16–34), and 19 (15–36) months, respectively (Fig. 1b–d).

### Relationship between low muscle mass and event occurrence

**Figure 2:**
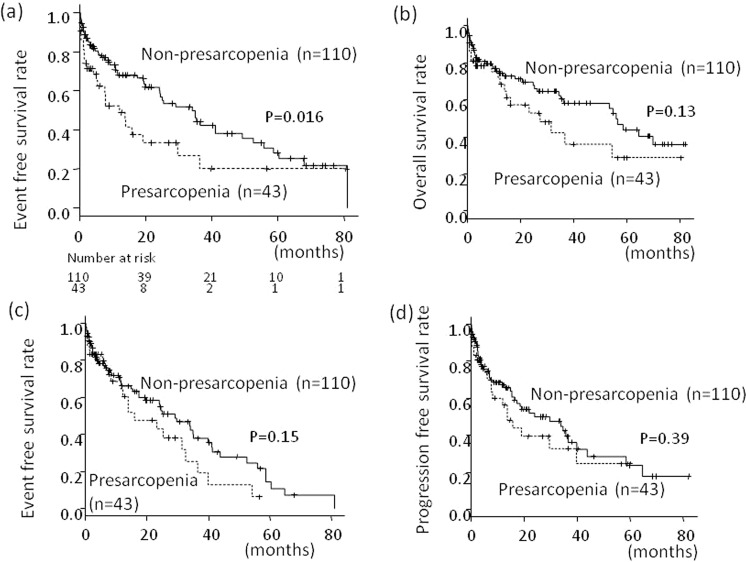
(**a**) Event-free survival (events necessary for hospitalization except recurrence of hepatocellular carcinoma), (**b**) overall survival, (**c**) event-free survival (events related to cirrhosis), (**d**) progression-free survival; patients with pre-sarcopenia versus patients without pre-sarcopenia.

Table 2 shows the patient background characteristics classified according to the presarcopenia status. The presarcopenia group (n = 43) had more males (93% vs. 67%, *P* = 0.001), lower body mass index (21 vs. 23, *P* = 0.008), more patients with ascites (33% vs. 15%, *P* = 0.02), larger maximum tumor diameter (63 vs. 43 mm, *P* = 0.004), and higher bilirubin levels (2.4 vs. 1.4 mg/dl, *P* = 0.05) at baseline compared to the group without presarcopenia (n = 110). The median event-free survival (12.0 months; range, 5.1–29.6 months) was significantly worse in the patients with presarcopenia than those without presarcopenia (34.1 months; range, 24.1–41.2 months; *P* = 0.016) (Fig. 2a). The event rates at 12, 36, and 60 months were 47%, 73%, and 80%, in the presarcopenia group and 32%, 56%, and 72%, respectively, in the group without presarcopenia. The median survival of the patients with presarcopenia tended to be shorter compared with patients without presarcopenia (27 vs. 56 months, *P* = 0.13) (Fig. 2b). The median time to cirrhosis-related event tended to be shorter in the patients with presarcopenia compared with those without presarcopenia (16 vs. 29 months, *P* = 0.15) (Fig. 2c). No significant difference was found in the median progression-free survival between the patients with and without presarcopenia (24 and 14 months, respectively; *P* = 0.39) (Fig. 2d).

**Table 2:** Comparison of backgrounds in patients with and without presarcopenia.

	With presarcopenia (n = 43)	Without presarcopenia (n = 110)	P value
Age: years, mean ± SD	72 ± 9.1	73 ± 9.2	0.48
Male: n (%)	40 (93)	74 (67)	0.001
BMI, mean ± SD	21 ± 3.2	23 ± 3.3	0.008
Complicated with diabetes: n (%)	12 (28)	32 (29)	1.0
Maximum size of HCC nodule in the liver: mm, median (range in IQR)	45 (25–111)	30 (18–60)	0.008
Number of HCC nodules: 1/2/3/4 or more, n (%)	22/9/4/8	59/16/10/25	0.89
Presence of ascites: n (%)	29 (67)	16 (15)	0.02
Presence of hepatic encephalopathy: n (%)	1 (2.3)	7 (6.4)	0.44
Child-Pugh grade: A/B/C: n (%)	23/15/5	72/30/8	0.36
HCC stage: 1/2/3/4: n (%)	4/16/12/11	22/46/20/22	0.25
JIS score: 0/1/2/3/4/5: n (%)	3/11/12/7/8/2	15/56/34/25/17/6	0.25
Albumin: g/dl, mean ± SD	3.4 ± 0.75	3.6 ± 0.56	0.14
Total bilirubin: g/dl, mean ± SD	2.4 ± 4.6	1.4 ± 1.7	0.046
γ-GTP: U/l, mean ± SD	157 ± 232	136 ± 177	0.56
Prothrombin time: %, mean ± SD	80 ± 22	83 ± 19	0.38
AFP: ng/ml, median (range in IQR)	48 (8.2–378)	29 (6.0–246)	0.22
AFP L3 index: %, median (range in IQR)	17 (3.2–54)	7.0 (2.0–31)	0.14
PIVKA-α: mAU/ml, median (range in IQR)	288 (32–11112)	79 (22–1848)	0.08
Taking BCAA: n (%)	10 (23)	33 (30)	0.68

Values are mean ± standard deviation. NASH; nonalcoholic steatohepatitis, BMI; body mass index, HCC; hepatocellular carcinoma, ALT; alanine aminotransferase, AFP; alpha-fetoprotein, AFP L3 fraction; Lens culinaris agglutinin-reactive fraction of AFP, PIVKA-II; prothrombin induced by vitamin K absence II.

### Relationship between the Child–Pugh grade and event occurrence

**Figure 3:**
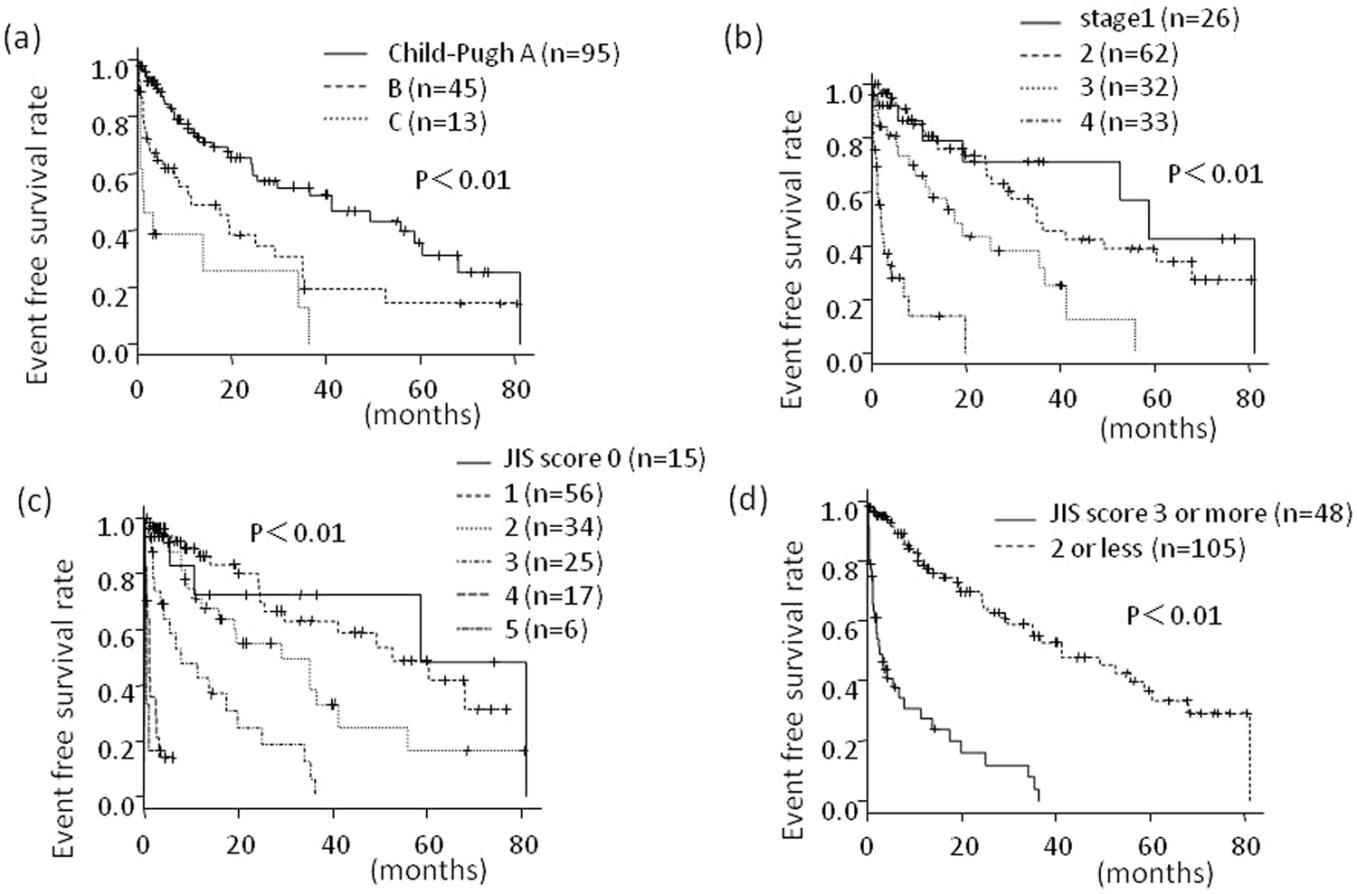
Prognostic factors for event-free survival (all events necessary for hospitalization). (**a**) Child-Pugh grade, (**b**) TNM staging, (**c**) JIS score, (**d**) JIS scores ≥ 3.

The median event-free survival was significantly worse in the patients with Child–Pugh grade C (1.1 months; range, 0.5–34.0 months) that those with Child–Pugh grade A (41.1 months; range, 24.2–60.3 months) or B (11.4 months; range, 4.1–29.2 months) (*P* < 0.01) (Fig. 3a). The event occurrence rates at 12, 36, and 60 months were 27%, 45%, and 64%, for Child–Pugh grade A; 51%, 82%, and 86%, for Child–Pugh grade B; and 62% 87%, and 100% for Child–Pugh grade C respectively.

### Relationship between TNM classification and event occurrence

The median event-free survival rates for patients with TNM stage 1, 2, 3, and 4 were 58.6 (19.4–not reached), 35.1 (24.5–62.9), 17.4 (7.8–36.6), and 2.0 (1.2–4.2) months (*P* < 0.01) (Fig. 3b). The event rates at 12, 36, and 60 months were 22%, 30%, and 57%, for stage 1; 19%, 52%, and 61%, for stage 2; 38%, 68%, and 100%, for stage 3; and 86%, 100%, and 100%, respectively, for stage 4.

### Relationship between the JIS score and event occurrence

The median event-free survival rates of the patients with JIS scores of 0, 1, 2, 3, 4, and 5 were 58.6 (5.6–not reached), 52.5 (25.5–not reached), 29.2 (12.0–41.1), 7.8 (3.5–19.8), 1.2 (0.4–2.7), and 0.46 (0.39–2.72) months, respectively (*P* < 0.01) (Fig. 3c). The event-free survival of the patients with JIS scores ≥ 3 (n = 48; median, 2.7 months; range 1.4–6.8 months) was significantly worse than those with JIS scores ≤ 2 (n = 105; median, 41.2 months; range, 29.2–60.4 months) (*P* < 0.01) (Fig. 3d). The event rates at 12, 36, and 60 months were 73%, 96%, and 100%, in those with JIS scores ≥ 3 and 21%, 45%, and 63%, in those with JIS scores ≤ 2, respectively.

### Factors related to event occurrence

Factors related to event occurrence were examined by univariate and multivariate analyzes (Table 3). The best cut-off values for JIS score, γ-GTP, AFP, and PIVKA-α were determined using ROC curve analysis. The best cut-off value was 3 for JIS score, 100 IU/L for γ-GTP, 200 ng/ml for AFP, and 200 mAU/ml for PIVKA-α. In univariate analysis, the following factors were significant: presence of presarcopenia, JIS score ≥3, γ-GTP ≥ 100 IU/L, AFP ≥ 200 ng/ml, and PIVKA-II ≥ 200 mAU/ml. In multivariate analysis, the presence of presarcopenia (hazard ratio [HR], 1.9), JIS score ≥3 (HR, 6.0), AFP ≥ 200 ng/ml (HR, 2.3), and PIVKA-II ≥ 200 mAU/ml (HR, 2.1) were significant factors associated with event occurrence. The cutoff values for γ-GTP, AFP, and PIVKA-II were obtained using the receiver-operating characteristic analysis determined by Youden index.

**Table 3:** Univariate and multivariable analysis of prognostic factors in patients with pre-sarcopenia.

Factors	Univariate		Multivariate			
	HR	95%CI	P value	HR	95% CI	P value
Age	1.0	0.99–1.0	0.23			
Male	1.5	0.86–2.5	0.16			
Presence of presarcopenia	1.8	1.1–2.9	0.02	1.9	1.1–3.4	0.03
JIS score > 3	6.3	3.8–10	<0.001	6.0	3.2–11	<0.001
γ-GTP > 100: U/l	1.8	1.1–2.8	0.02	1.0	0.61–1.8	0.89
AFP > 200: ng/ml	4.4	2.6–7.5	<0.001	2.3	1.2–4.5	0.02
AFP L3 index > 10: %	1.6	0.92–2.7	0.09			
PIVKA-α > 200: mAU/ml	3.4	2.1–5.7	<0.001	2.1	1.1–3.8	0.02

HR; hazard ratio, CI; confidence interval, AFP; alpha-fetoprotein, AFP L3 fraction; Lens culinaris agglutinin-reactive fraction of AFP, PIVKA-II; prothrombin induced by vitamin K absence II.

### Relationship between low muscle mass and event occurrence after stratification by the JIS score

**Figure 4:**
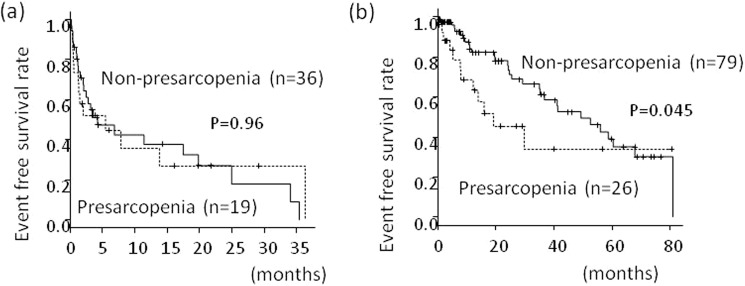
(**a**) Presarcopenia and event-free survival in patients with JIS score of 3 or more, (**b**) Presarcopenia and event-free survival in patients with JIS score of 2 or less.

The frequency of presarcopenia in the patients with JIS scores ≥3 was 35%, which was not different with that in the patients with JIS scores ≤2 (25%) *(P* = 0.18). Table 4 shows the patient background characteristics classified according to the presence of presaropenia and JIS score. Among the patients with JIS scores ≥3, there was no difference in the event occurrence rates in those with and without presarcopenia (*P* = 0.96) (Fig. 4a). Among the patients with JIS scores ≤2, the median event-free-survival was significantly shorter in those with presarcopenia (19.1 months; range, 7.8 months–not reached) compared with those without presarcopenia (49.2 months; range, 35.0–67.9 months) (*P* = 0.045) (Fig. 4b).

**Table 4:** Comparison of backgrounds in patients classified according to the presence of presaropenia and JIS score.

	With presarcopenia and JIS score >3 (n = 17)	With presarcopenia and JIS score <2 (n = 26)	Without presarcopenia and JIS score >3 (n = 31)	Without presarcopenia and JIS score <2 (n = 79)
Age: years, mean ± SD	68 ± 8.0	75 ± 8.6	71 ± 10	75 ± 8.6
Male: n (%)	15 (88)	25 (96)	25 (81)	49 (62)
BMI, mean ± SD	20 ± 3.2	22 ± 3.2	22 ± 3.6	23 ± 3.1
Complicated with diabetes: n (%)	6 (35)	6 (23)	8 (26)	24 (30)
Maximum size of HCC nodule in the liver: mm, median (range in IQR)	105(50–120)	29(20–52)	67(40–95)	25(15–41)
Number of HCC nodules: 1/2/3/4 or more, n	7/3/0/7	15/6/4/0	7/8/2/14	55/8/8/8
Presence of ascites: n (%)	9(53)	5(19)	12(39)	4(5)
Presence of hepatic encephalopathy: n (%)	1(6)	0(0)	4(13)	3(4)
Child-Pugh grade: A/B/C: n	2/10/5	22/2/2	10/15/6	59/19/1
HCC stage: 1/2/3/4: n	0/2/4/11	4/14/8/0	0/1/8/22	22/45/12/0
JIS score: 0/1/2/3/4/5: n	0/0/0/7/8/2	3/11/12/0/0/0	0/0/0/18/9/4	12/45/22/0/0/0
Albumin: g/dl, mean ± SD	2.9 ± 0.61	3.8 ± 0.64	3.3 ± 0.54	3.7 ± 0.52
Total bilirubin: g/dl, mean ± SD	2.8 ± 0.68	0.89 ± 0.37	2.4 ± 2.9	1.0 ± 0.57
γ-GTP: U/l, mean ± SD	285 ± 318	69 ± 70	257 ± 265	94 ± 108
Prothrombin time: %, mean ± SD	72 ± 22	86 ± 19	76 ± 19	86 ± 18
AFP: ng/ml, median (range in IQR)	284(17–28457)	39(6.1–127)	803(37–7781)	13(4.8–57)
AFP L3 index: %, median (range in IQR)	54(25–74)	6.8(0.5–28)	30(6.2–62)	4.6(0.5–12)
PIVKA-α: mAU/ml, median (range in IQR)	9618(6251–24752)	99(24–1702)	2018(125–17501)	45(20–314)
Taking BCAA: n (%)	6(35)	4(15)	11(36)	22(28)

Values are mean ± standard deviation. NASH; nonalcoholic steatohepatitis, BMI; body mass index, HCC; hepatocellular carcinoma, ALT; alanine aminotransferase, AFP; alpha-fetoprotein, AFP L3 fraction; Lens culinaris agglutinin-reactive fraction of AFP, PIVKA-II; prothrombin induced by vitamin K absence II.

## Discussion

The frequency of presarcopenia in this study cohort was 28%, and presarcopenia was an important factor in predicting event occurrence requiring hospitalization in patients with hepatocellular carcinoma (HCC). Sarcopenia is characterized by muscle weakness and low muscle mass^2^. According to the assessment criteria for sarcopenia in liver disease (first edition), patients with chronic liver disease who have decreased grip strength (<26 kg in males and <18 kg in females) and low muscle mass by CT (<42 cm²/m² in males and <38 cm²/m² in females) or BIA (<7 kg/m² in males and <5.7 kg/m² in females), are diagnosed with sarcopenia^1^. In Japanese patients with liver cirrhosis or liver cancer, 11%–68% are diagnosed with sarcopenia. Sarcopenia was shown to be associated with prognosis, cancer development, encephalopathy, decreased quality of life, decreased performance status, bone fracture, sepsis, and early hospitalization^4,14–16^. However, the mechanism why sarcopenia causes poor prognosis is not clear^17^. Presarcopenia refers to patients with low muscle mass regardless of muscle weakness, and was demonstrated to be a poor prognostic factor in patients undergoing liver transplantation, surgery, and sorafenib treatment^18–21^. It is important to note that presarcopenia was shown to be a prognostic factor independent of liver function and HCC staging.

The rate of event occurrence was significantly higher in the patients with JIS score ≥3 than those with JIS score ≤2. The JIS score is a good prognostic factor. Accurate prediction of prognosis in the patients with HCC is extremely important for selection of appropriate treatment in each case. The prognosis of patients with HCC depends not only on cancer staging but also on liver function; therefore, the prediction is more complicated in HCC compared with cancers in other organs. There are several classifications utilized for HCC patients, and the best prediction system remains controversial^22–31^. The JIS score developed by the Japan Hepatocarcinoma Research Association, combines the Child–Turcotte–Pugh classification with the TNM classification. The JIS score is an objective prediction method that can be implemented easily in clinical settings^32^. The stratification as well as prognostic prediction using the JIS score were shown to be equivalent or superior to the Cancer of the Liver Italian Program score^26^. The JIS score can accurately classify liver cancer from early to advanced stage, therefore is considered to be useful for examining various cases of HCC as in this study. In the present study, the JIS score was used to accurately stratify various cases and was an outstanding prognostic predictor.

The frequencies of presarcopenia were 35% and 25% in the patients with JIS scores of ≥3 and ≤2, respectively, which were not significantly different. Importantly, among the patients with JIS scores ≥ 3, indicating relatively poor liver function and HCC status, there was no difference in the event occurrence rates between those with and without presarcopenia; however, among the patients with JIS scores ≤ 2, which indicated relatively good liver function and HCC status, the prognosis of the patients with presarcopenia was significantly worse than those without presarcopenia. This is the first study to report the relationship between the JIS score and presarcopenia. We have thought that hepatic reserve and tumor conditions are the most important prognostic factors in patients with HCC, but muscle wasting is also an important factor as a marker to reflect general condition. In HCC patients with high JIS scores, presarcopenia progresses to irreversible cachexia, and there are many cases where improving metabolic dysfunction and prognosis after treatment is difficult. Conversely, in patients with low JIS scores, treatment intervention may prevent presarcopenia and reduce the event occurrence rate. Studies are increasingly investigating treatment of sarcopenia, and intervention of sarcopenia with diet, exercise therapy, and medication were shown to slow the depletion rate of muscle mass and improved prognosis^9,10,33^. It is necessary to select patients whose diagnosis can be improved by preventing presarcopenia, and initiate appropriate interventions. Our present study suggested that although hepatic reserve and tumor conditions are the most important factors in patients with HCC, presarcopenia is also an important factor.

Poor prognostic factors other than presarcopenia and a JIS score ≥ 3 were γ-GTP ≥ 100 IU/L, AFP ≥ 200 ng/ml, and PIVKA-II ≥ 200 mAU/ml. γ-GTP reflects systemic inflammation and was reported to be a prognostic marker for many cancer types, similar to the neutrophil-lymphocyte ratio and platelet-lymphocyte ratio^34–36^. γ-GTP, which plays an important role in glutathione metabolism, was shown to be related to apoptosis of normal hepatocytes, oxidative stress, and carcinogenesis^37,38^. Recent studies reported that the prognosis was worse in patients with high γ-GTP levels compared to those with low γ-GTP levels among those undergoing hepatectomy, radiofrequency ablation, or transcatheter arterial embolization^39–41^. In this study, the event occurrence rate was significantly higher in the patients with high γ-GTP levels compared to those with low γ-GTP levels. Males were previously reported to have physiologically higher levels of γ-GTP^42,43^, and it is necessary to analyze the relationship of γ-GTP with sex and the molecular mechanism in patients with HCC in future studies.

The current study has several limitations. This was a retrospective, single-center study. The estimation of statistical power and setting the sample size were not carried out before registration. The number of patients was small in our hospital, and sufficient statistical power was not obtained (statistical power of this study was 0.62). Hence, multi-center randomized study with a large sample will be more persuasive. Grip strength was not measured, and evaluation of muscle mass was based on PMI and simplified PMI, which does not reflect the general condition of the patient. Hepatic background characteristics were not unified. Additionally, the cohort included a high frequency of male patients, and analyses based on sex were not performed.

To the best of our knowledge, this is the first study that examines the relationship of presarcopenia, the JIS score, and event occurrence in patients with primary HCC. Presarcopenia, similar to liver function and HCC staging, was an important prognostic factor. Particularly, in patients with low JIS scores, treatment intervention may prevent presarcopenia and improve prognosis. Early diagnosis and treatment of presarcopenia are necessary in patients with HCC, and it will be necessary to clear which cases should be evaluated the presence of sarcopenia, and consider the criteria, judgment timing, and the therapeutic intervention.

## Conclusion

Low muscle mass is associated with event occurrence rate requiring hospitalization in patients with primary hepatocellular carcinoma. In patients with JIS scores ≤2, the prognosis of those with presarcopenia was significantly worse than those without presarcopenia.
